# Frequency-Specific EEG burst dynamics in Parkinson’s Disease and freezing of gait

**DOI:** 10.1007/s00415-026-14149-6

**Published:** 2026-09-23

**Authors:** Matthew Leedom, Md Rezwanul Akter Pallab, Vishal Bharmauria, Oliver Flouty, Samuel Stuart, Martina Mancini, Arun Singh

**Affiliations:** 1 Biomedical and Translational Sciences, Sanford School of Medicine, University of South Dakota, 414 E. Clark St., Vermillion, SD 57069 USA; 2 Department of Physical Therapy, School of Health Science, University of South Dakota, Vermillion, SD USA; 3 The Tampa Human Neurophysiology Lab, Department of Neurosurgery and Brain Repair, Morsani College of Medicine, University of South Florida, Tampa, FL USA; 4 Department of Neurology, Oregon Health & Science University, Portland, OR USA; 5 Department of Sport, Exercise and Rehabilitation, Northumbria University, Newcastle Upon Tyne, NE1 8ST UK; 6 Department of Neuroscience, Sanford School of Medicine, University of South Dakota, Sioux Falls, SD USA; 7 Center for Vision Research & Center for Integrative and Applied Neuroscience,, York University, Toronto, Ontario Canada

**Keywords:** Parkinson’s disease, Freezing of gait, Bursts, EEG, Oscillations

## Abstract

**Background:**

Parkinson’s disease (PD) disrupts motor networks, but conventional EEG spectral analyses may miss the transient, burst-like structure of pathological oscillations. Freezing of gait (FOG) is a disabling and poorly understood gait disturbance that may involve altered cognitive-motor control. This study aims to determine whether resting-state electroencephalography burst dynamics provide frequency-specific markers of PD and FOG.

**Methods:**

Resting-state EEG was analyzed from 237 participants across three study sites, including 88 healthy controls and 149 individuals with PD assessed in the clinically defined ON-medication state. Participants with PD were also classified as having FOG (PDFOG +) or not having FOG (PDFOG −). Analyses were harmonized across sites using 11 common EEG channels. Burst dynamics were quantified in theta, alpha, low-beta, and high-beta bands using amplitude-envelope thresholding. Primary analyses focused on low- and high-beta burst dynamics at mid-frontal lead for healthy controls vs PD comparisons, and theta, low-beta, and high-beta burst dynamics for healthy control, PDFOG−, and PDFOG+ comparisons. FOG severity associations were examined using a study site–standardized FOGQ z-scores.

**Results:**

People with PD showed altered low-beta burst timing, characterized by more frequent, but shorter low-beta bursts compared with healthy controls. These effects were evident at the mid-frontal region and supported by exploratory topographic maps. In three-group analyses, low-beta burst rate and duration differed across groups, but adjusted PD-only models did not support robust PDFOG+ vs PDFOG− differences. In contrast, FOG severity was most consistently associated with theta amplitude-related burst metrics.

**Conclusions:**

Resting-state EEG burst dynamics reveal dissociable oscillatory signatures in PD. Low-beta burst timing abnormalities reflect general PD-related motor network dysfunction, whereas theta burst amplitude may relate to FOG severity and cognitive-motor network burden.

**Supplementary Information:**

The online version contains supplementary material available at https://doi.org/10.1007/s00415-026-14149-6.

## Introduction

Parkinson’s disease (PD) is a progressive neurodegenerative disorder characterized by bradykinesia, rigidity, tremor, and impairments in gait and postural control. Although dopaminergic therapy improves many cardinal motor symptoms, gait dysfunction and freezing of gait (FOG) frequently persist as disabling and therapeutically challenging manifestations [1, 2]. FOG is defined as a brief, episodic inability to generate effective stepping despite the intention to walk and is associated with falls, reduced mobility, loss of independence, and diminished quality of life [3]. The mechanisms underlying FOG remain incompletely understood. Current models, however, suggest that FOG reflects abnormal interactions across motor, cognitive, attentional, and basal ganglia–cortical control networks, rather than dysfunction confined to a single isolated circuit [4, 5].

Neural oscillations provide an important framework for understanding network-level dysfunction in PD. Beta-band activity, in particular, has been strongly implicated in PD motor pathophysiology [6, 7]. Excessive beta synchronization within basal ganglia–cortical motor circuits has been associated with impaired movement initiation, bradykinesia, rigidity, and reduced motor flexibility [7–9]. Both dopaminergic therapy and deep brain stimulation (DBS) suppress pathological beta activity, and reductions in beta oscillatory power have been linked to clinical motor improvement [10, 11]. These findings support the view that abnormal beta-band synchronization represents a key physiological marker of impaired motor network function in PD.

Traditionally, EEG and local field potential studies have quantified oscillatory activity using average spectral power within predefined frequency bands. However, average power may obscure the inherently transient nature of oscillatory activity [12]. Beta activity does not occur as a continuously sustained signal; rather, it is expressed as brief, intermittent events, commonly referred to as “bursts” [13, 14]. Burst-based analyses, therefore, offer a more physiologically meaningful characterization of pathological network synchronization than average power alone. In PD, beta burst duration and burst dynamics have been shown to vary with medication state and degree of motor impairment, and modulation of beta bursts has been linked to motor performance as well as to the effects of DBS [15, 16].

Several burst characteristics may be mechanistically relevant to PD motor dysfunction, including the frequency with which bursts occur, their duration, their amplitude, and the overall proportion of time the signal spends in a burst state. Prolonged or more frequent beta bursts may reflect reduced flexibility of sensorimotor networks and could impair movement initiation or task switching [11, 15, 16]. Accordingly, burst metrics, including burst rate, burst duration, burst amplitude, and time in burst, may provide complementary and potentially more sensitive information about the temporal organization of beta activity in PD than average power measures.

Although beta activity is central to PD motor physiology, FOG may involve additional frequency-specific abnormalities extending beyond the beta band. FOG episodes are most likely to emerge in situations requiring heightened attentional control, conflict monitoring, turning, dual-tasking, or rapid motor adaptation, implicating cognitive-motor control networks in its pathophysiology [4, 17]. Theta-band activity, particularly within the mid-frontal region, has been linked to cognitive control and conflict monitoring, and abnormal theta oscillations have been reported during the temporal evolution of FOG episodes in PD [18, 19]. Functional neuroimaging studies further indicate that FOG is associated with altered interactions between cognitive control networks and basal ganglia circuits [4, 20]. Taken together, these findings suggest that theta burst dynamics may be especially relevant to cognitive-motor dysfunction and FOG, whereas low- and high-beta burst dynamics may predominantly reflect motor network abnormalities associated with PD and gait impairment more broadly.

Despite growing interest in oscillatory burst patterns, relatively little is known about how cortical EEG burst dynamics differ between healthy controls and individuals with PD, or how burst dynamics are modulated by the presence of FOG [12]. Most prior EEG studies have focused on average spectral power, functional connectivity, or event-related changes, whereas few studies have systematically examined burst-based metrics across multiple frequency bands [21–24]. It also remains unclear whether burst abnormalities are spatially localized to central sensorimotor regions or more broadly distributed across frontal, central, parietal, and occipital cortices. A harmonized, multi-center EEG dataset offers a valuable opportunity to investigate these questions in a larger and clinically diverse PD sample.

This study examined EEG burst dynamics in healthy controls and individuals with PD across three sites using a harmonized 11-channel montage spanning frontal to occipital regions; all PD participants with PD were assessed in the clinically defined ON-medication state. Burst detection was performed in theta, alpha, low-beta, and high-beta bands, with primary metrics capturing burst rate, duration, amplitude, and time in burst, and secondary metrics indexing prolonged bursts, high-amplitude bursts, and temporal variability [15, 25, 26]. We first assessed whether beta-band burst dynamics differed between healthy controls and participants with PD, focusing on low- and high-beta activity at the mid-frontal electrode, given its relevance to PD motor pathophysiology [19, 27, 28]. We then evaluated differences across controls, participants with PD who do not have FOG (PDFOG −), and participants with PD who do have FOG (PDFOG +), with emphasis on theta and beta bands. Finally, exploratory topographic analyses examined the spatial distribution of burst-related group differences. Together, these analyses tested whether burst-based EEG metrics provide frequency-specific and spatially distributed markers of PD and FOG-related cortical network dysfunction.

## Methods

### Participants and clinical assessment

Resting-state EEG data were obtained from 237 participants across three sites: the University of Iowa (site 1), the University of South Dakota (site 2), and Oregon Health & Science University (site 3). The sample included 149 individuals with PD and 88 healthy controls. Participants with PD were classified as PDFOG+ or PDFOG−, yielding 73 PDFOG+ and 76 PDFOG− participants.

Site-specific distributions were: site 1 (83 PD, 41 healthy controls; 42 PDFOG+, 41 PDFOG −), site 2 (32 PD, 27 healthy controls; 15 PDFOG+, 17 PDFOG −), and site 3 (34 PD, 20 healthy controls; 16 PDFOG+, 18 PDFOG −). Portions of site 1 data have been reported previously [27, 29–31]. All PD participants were tested in the clinically defined ON-medication state while taking their usual dopaminergic medication regimen. ON-medication testing was used as part of the harmonized multi-site protocol to improve safety, tolerability, and consistency across participants [19, 32]. The ON-medication state was defined clinically, with participants assessed during their typical medicated functional state. The timing of the last dopaminergic medication dose relative to EEG acquisition was not uniformly standardized or recorded across all participants and study sites.

The study procedures were approved by the Institutional Review Boards of the participating institutions: the University of Iowa Institutional Review Board, the University of South Dakota Institutional Review Board, and the Oregon Health & Science University Institutional Review Board. All procedures were conducted in accordance with the Declaration of Helsinki. Participants provided written informed consent. Motor severity was assessed using MDS-UPDRS III [33], cognition using the Montreal Cognitive Assessment [34], and FOG status using self-report, questionnaire measures, and clinical observation during gait assessment, consistent with prior criteria [19, 35]. Sites 1 and 2 used the Freezing of Gait Questionnaire (FOGQ [36]), whereas Site 3 used the New Freezing of Gait Questionnaire (NFOG-Q [37]). When available, clinician-observed freezing during the standardized gait assessment was also used to support PDFOG+ classification.

Because FOG severity differed across sites, scores were standardized within site prior to the pooled analyses. Demographic and clinical characteristics for the full cohort are summarized in Table 1, and site-specific demographic information is provided in Table S1.

**Table 1:** Demographics and clinical characteristics

Measure	HC(*n=*88)	PD (*n=*149)	PDFOG− (*n=*76)	PDFOG+ (*n=*73)	HC vs. PD	PDFOG− vs. PDFOG+	HC vs. PDFOG− vs. PDFOG+
Gender (M/F)	42/46	98/51	46/30	52/21	–	–	
Age (years)	69.0 ± 0.8	69.4 ± 0.6	68.9 ± 0.8	69.8 ± 1.0	*p = *0.742	*p = *0.487	*p = *0.744
DD (years)	–	5.6 ± 0.3	4.5 ± 0.4	6.8 ± 0.5	–	p < 0.001**	–
LEDD (mg)	–	757.9 ± 38.9	615.0 ± 50.1	910.9 ± 54.8	–	p < 0.001**	–
MDS-UPDRS III	–	20.8 ± 1.2	16.1 ± 1.4	25.6 ± 1.9	–	p < 0.001**	–
MoCA	26.9 ± 0.2	25.2 ± 0.3	26.0 ± 0.3	24.5 ± 0.5	p < 0.001**	p = 0.012*	p < 0.001**

Data are presented as mean ± SEM. Group differences between HC and participants with PD were evaluated using independent t-tests. Group differences between PDFOG− and PDFOG+ were also evaluated using independent t-tests. Comparisons among HC, PDFOG**–**, and PDFOG+ groups were evaluated using one-way ANOVA

*DD* Disease duration, *LEDD* Levodopa equivalent daily dose, *MDS-UPDRS III* motor Unified Parkinson’s Disease Rating Scale, *MoCA* Montreal Cognitive Assessment, *HC* healthy controls, *PD* Parkinson’s disease, *PDFOG* + people with PD who have FOG, *PDFOG−* people with PD who do not have FOG

Statistical significance for t-tests and ANOVA presented as *p *value. *p<0.05; **p<0.01

### EEG recordings and processing

Resting-state EEG was acquired using site-specific systems. Sites 1 and 2 used a 64-channel and 96-channel Brain Vision system (sampling rate 500 Hz), respectively. Site 3 used a 32-channel TMSi Mobita system (sampling rate 2000 Hz). Recordings were obtained under resting conditions while participants were instructed to remain still and minimize eye, head, and limb movements.

To harmonize datasets, analyses were restricted to a common 11-channel montage (F3, Fz, F4, C3, Cz, C4, P3, Pz, P4, O1, O2), spanning frontal through occipital regions. Primary analyses focused on the mid-frontal Cz electrode due to its relevance to motor and gait-related activity [19, 27, 28, 30, 38]. The focus on Cz was also based on both methodological and physiological considerations. Cz was consistently available across all study sites, enabling harmonized analysis across acquisition systems. Although Cz is conventionally classified as a midline central or vertex electrode in the 10–20 system, we use the term “mid-frontal Cz” operationally to refer to a midline fronto-central EEG signal, consistent with our previous work [19, 27, 35, 39]. In the present study, Cz, therefore, provided a practical and physiologically relevant midline electrode for examining oscillatory burst dynamics related to PD motor-network dysfunction and FOG severity.

EEG preprocessing was performed in MATLAB using EEGLAB [40]. Data were epoched into 3-s segments, re-referenced to the average, and baseline corrected. Artifacts were removed using a pipeline incorporating FASTER [41] and ADJUST [42], and CleanLine. Bad channels and epochs were identified and corrected or rejected, and independent component analysis (runica) was used to remove artifact components. Cleaned data were restricted to the 11-channel montage, and burst analyses were performed using custom MATLAB routines after removal of artifactual components.

#### Burst detection

Burst detection was performed separately for each participant, channel, and frequency band. The analyzed bands were theta (4–8 Hz), alpha (8–13 Hz), low-beta (13–20 Hz), and high-beta (20–30 Hz). For each band, the signal was filtered within the relevant frequency range, and bursts were defined as periods in which the amplitude envelope exceeded the 75th percentile threshold for that subject, channel, and band.

The primary burst metrics were burst rate (bursts/min), median burst duration (s), median burst amplitude (a.u.), and time in burst (% of analyzed recording time). The secondary burst metrics were 90th percentile burst duration (s), 90th percentile burst amplitude (a.u.), inter-burst interval coefficient of variation, and burstiness index. Primary metrics were selected to capture typical burst frequency, duration, amplitude, and occupancy, whereas secondary metrics were used to characterize prolonged or high-amplitude bursts, variability in burst timing, and overall burstiness.

### Statistical analysis

#### Healthy controls vs PD

Primary analyses compared healthy controls and participants with PD for low-beta and high-beta-band bursts at mid-frontal Cz using subject-level metrics. For each beta sub-band, Benjamini–Hochberg false discovery rate (FDR) correction was applied across the four predefined primary burst metrics.

Exploratory analyses were also performed across all 11 EEG channels. For each band and burst metric, healthy control versus PD group differences were tested at each channel. Topographic difference maps were generated, and FDR correction was applied across channels.

#### Healthy controls vs PDFOG− vs PDFOG+

Three-group comparisons were conducted across theta, low-beta, and high-beta bands using Kruskal–Wallis tests with pairwise comparisons, as appropriate. The primary channel-level analysis was performed at mid-frontal Cz using the four primary burst metrics, with parallel secondary analyses using the four secondary burst metrics.

The primary PD-specific FOG contrast was tested within PD participants only using a linear model adjusted for disease duration and MDS-UPDRS III score (Burst metric ~ FOG group + disease duration + UPDRS III). FDR correction was applied separately within each frequency band across the four primary metrics and separately across the four secondary metrics.

### Topographic visualization

Topographic maps were generated across the 11 common EEG channels to visualize spatial patterns of group differences. For control versus PD and FOG subgroup analyses, difference maps were computed, with uncorrected (*p < *0.05) and FDR–corrected results indicated using standardized markers.

### FOG severity correlation analysis

FOG severity was standardized within the site (FOGz = (FOG score − center mean FOG score)/center SD) and pooled across participants with PD. Spearman correlations were used to examine associations between mid-frontal Cz burst metrics and FOG severity z-scores for theta, low-beta, and high-beta bands. Primary correlation analyses used the four primary burst metrics, and secondary analyses used the four secondary burst metrics.

Partial Spearman correlations were additionally performed while adjusting for disease duration and UPDRS III. FDR correction was applied within each frequency band across the four predefined metrics.

## Results

### Healthy controls vs PD: primary mid-frontal analyses

**Fig. 1:**
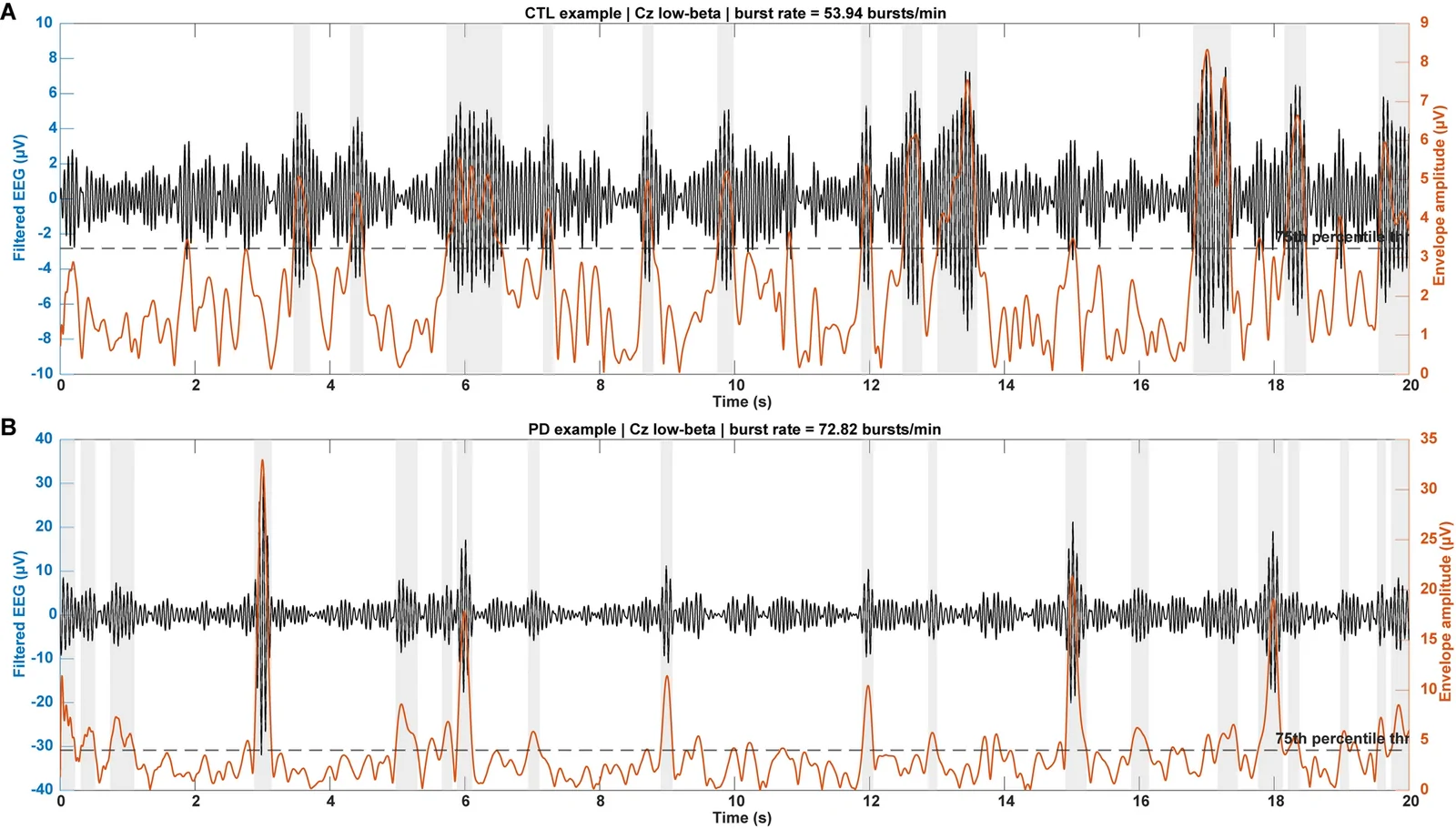
Example low-beta burst detection in a control and a PD participant. Example low-beta Mid-frontal ‘Cz’ traces are shown from one healthy control participant (**A**) and one participant with PD (**B**). Participants were selected based on proximity to their respective group median low-beta Cz burst profile across the four primary burst metrics. For each example, the bandpass-filtered low-beta signal, amplitude envelope, detection threshold, and detected burst periods are shown. Shaded regions indicate periods classified as bursts. These examples are intended to illustrate the burst detection procedure and were not used for statistical inference

Figure 1 illustrates burst detection at the mid-frontal Cz electrode, highlighting representative low-beta burst profiles from a control and a participant with PD. In the mid-frontal region, participants with PD exhibited significant alterations in low-beta burst dynamics. Specifically, burst rate was higher compared with healthy controls (*p = *0.002; FDR *p = *0.004), while median burst duration was shorter (*p = *0.002; FDR *p = *0.004; Table 2). In contrast, low-beta median burst amplitude and time in burst did not differ between groups (amplitude: *p = *0.485, FDR *p = *0.618; time in burst: *p = *0.618, FDR *p = *0.618). In the high-beta band, none of the primary metrics reached significance after correction (all FDR *p* ≥ 0.173). High-beta time in burst was lower in participants with PD at the uncorrected level (*p = *0.014), but this effect did not remain significant after correction (FDR *p = *0.055). Together, these results indicate that participants with PD, assessed in the clinically defined ON-medication state, showed more frequent and shorter low-beta bursts at the mid-frontal level, without consistent changes in burst amplitude or overall burst occupancy.

**Table 2:** Primary mid-frontal Cz burst metrics in healthy controls vs. PD

Metric	Low-beta HC (*n =* 88)	Low-beta PD (*n =* 149)	p	pFDR	High-beta HC (*n =* 88)	High-beta PD (*n =* 149)	*p*	pFDR
Burst rate (burst/min)	64.30 ± 0.31	65.54 ± 0.24	0.002**	0.004**	66.24 ± 0.26	65.69 ± 0.23	0.087	0.173
Median burst duration(s)	0.19 ± 0.001	0.18 ± 0.001	0.002**	0.004**	0.15 ± 0.001	0.15 ± 0.001	0.281	0.375
Median burst amplitude (a.u.)	6.32 ± 0.83	5.53 ± 0.51	0.485	0.618	7.12 ± 1.02	7.28 ± 0.84	0.381	0.381
Time in burst (%)	22.13 ± 0.04	22.14 ± 0.04	0.618	0.618	18.59 ± 0.09	18.34 ± 0.08	0.014*	0.055

Data are presented as mean ± SEM. Group differences between HC and participants with PD were evaluated using independent t-tests. Reported p-values are uncorrected. pFDR values represent Benjamini–Hochberg false discovery rate-adjusted p-values corrected within each frequency band across burst metrics

Statistical significance was defined as *pFDR < 0.05, **pFDR < 0.01

*HC* healthy controls, *PD* Parkinson disease

### Healthy controls vs PD: topographic patterns

Topographic maps (Fig. S1A) demonstrated that low-beta abnormalities extended beyond the mid-frontal region, with higher burst rate and shorter duration across multiple central and posterior channels in participants with PD. In contrast, differences in amplitude and time in burst were weaker and spatially localized. In the high-beta band (Fig. S1B), group differences were less consistent, with localized reductions in time in burst that paralleled the uncorrected mid-frontal findings, but that did not show robust corrected effects.

Topographic maps for theta and alpha bands (Fig. S2) showed less consistent group differences, indicating that the strongest and most spatially coherent healthy control versus PD effects were specific to the low-beta frequency range.

### Healthy controls vs PD: secondary metrics

Secondary metrics analyses (Table S2) did not reveal additional significant differences after correction. In low-beta, 90th percentile burst duration was shorter in participants with PD at the uncorrected level (*p = *0.021; FDR *p = *0.084), while other metrics, including amplitude, inter-burst interval variability, and burstiness index, were not significant (all FDR p ≥ 0.206). In high-beta, none of the secondary metrics differed between groups (all FDR p ≥ 0.528). Topographic maps (Fig. S3) showed localized effects across bands, but these were less consistent than the primary low-beta findings.

### Healthy controls vs PDFOG− vs PDFOG+: primary analyses

Three-group analyses at the mid-frontal Cz location (Table 3) showed significant differences in low-beta burst timing. Burst rate differed across groups (*p = *0.005; FDR *p = *0.010), and median burst duration was also significant (*p = *0.005; FDR *p = *0.010). However, amplitude and time in burst were not different (amplitude: *p = *0.591, FDR *p = *0.674; time in burst: *p = *0.674, FDR *p = *0.674). In the theta band, median burst amplitude showed a group effect before correction (*p = *0.014), but this did not remain significant after FDR adjustment (FDR *p = *0.056). Other theta metrics were not significant (all FDR p ≥ 0.756). In the high-beta band, no metrics were significant after correction. Time in burst showed an uncorrected group effect (*p = *0.05), but this did not persist after correction (FDR *p = *0.205).

**Table 3:** Primary mid-frontal Cz burst metrics across healthy controls, PDFOG−, and PDFOG+ groups

Metric	HC (*n =* 88)	PDFOG− (*n =* 76)	PDFOG+ (*n =* 73)	*p*	pFDR	Adjusted β (PDFOG− vs. PDFOG +)	*p*	Adjusted pFDR
**Theta**								
Burst rate (burst/min)	51.65 ± 0.23	51.76 ± 0.37	51.48 ± 0.30	0.756	0.756	−0.219	0.671	0.671
Median burst duration(s)	0.27 ± 0.002	0.26 ± 0.002	0.27 ± 0.002	0.462	0.756	0.002	0.626	0.671
Median burst amplitude	8.54 ± 3.82	5.12 ± 0.66	5.77 ± 0.69	0.014	0.056	−0.613	0.540	0.671
Time in burst (%)	23.92 ± 0.02	23.95 ± 0.03	23.95 ± 0.02	0.648	0.756	−0.026	0.504	0.671
**Low-beta**								
Burst rate (burst/min)	64.30 ± 0.31	65.33 ± 0.36	65.82 ± 0.32	0.005*	0.010*	0.433	0.414	0.898
Median burst duration(s)	0.19 ± 0.001	0.18 ± 0.001	0.18 ± 0.001	0.005*	0.010*	0.000	0.898	0.898
Median burst amplitude	6.32 ± 0.83	5.17 ± 0.71	5.95 ± 0.73	0.591	0.674	0.374	0.733	0.898
Time in burst (%)	22.13 ± 0.04	22.11 ± 0.05	22.16 ± 0.06	0.674	0.674	0.040	0.627	0.898
**High-beta**								
Burst rate (burst/min)	66.24 ± 0.26	65.63 ± 0.35	65.75 ± 0.31	0.197	0.393	−0.009	0.986	0.986
Median burst duration(s)	0.15 ± 0.001	0.15 ± 0.001	0.15 ± 0.001	0.435	0.574	0.000	0.787	0.986
Median burst amplitude	7.12 ± 1.02	6.67 ± 1.02	7.98 ± 1.35	0.574	0.574	1.150	0.527	0.986
Time in burst (%)	18.59 ± 0.09	18.32 ± 0.11	18.36 ± 0.11	0.051	0.205	0.046	0.782	0.986

Data are presented as mean ± SEM of participant-level burst metrics. Group differences were assessed using Kruskal–Wallis tests. Reported *p*-values are uncorrected. pFDR values represent Benjamini–Hochberg false discovery rate-adjusted* p*-values corrected within each frequency band across burst metrics. β coefficients represent the estimated PDFOG+ versus PDFOG− effect adjusted for disease duration and MDS-UPDRS III score. Adjusted pFDR values represent false discovery rate-adjusted p-values for the adjusted models

Statistical significance was defined as *pFDR < 0.05

*HC* healthy control, *PDFOG−* PD without freezing of gait, *PDFOG*+ PD with freezing of gait, *MDS-UPDRS III* Movement Disorder Society Unified Parkinson's Disease Rating Scale Part III

Importantly, PD-only adjusted models showed no significant differences between the PDFOG+ and PDFOG− groups for any primary burst metrics after controlling for disease duration and MDS-UPDRS III. This indicates that these effects were not specific to FOG subgroup status.

### Healthy controls vs PDFOG− vs PDFOG+: topographic analyses

Topographic maps (Fig. S4) reinforced the channel-level findings. Theta maps (Fig. S4A) showed amplitude-related reductions in PD groups relative to healthy controls, although these effects were not robust in adjusted analyses. In low-beta bands (Fig. S4B), both PDFOG− and PDFOG+ groups exhibited higher burst rates and shorter burst duration relative to healthy controls across several channels, with some effects surviving FDR correction. High-beta maps (Fig. S4C) were spatially heterogeneous and showed weaker group differences. Overall, FOG subgroup contrasts were limited and less spatially consistent than the healthy control vs PD effects.

### Secondary subgroup analyses

Secondary mid-frontal analyses (Table S3) did not reveal significant group differences after correction. Theta 90th percentile amplitude showed an uncorrected effect (*p = *0.021; FDR *p = *0.086), and low-beta 90th percentile duration showed a similar trend (*p = *0.032; FDR *p = *0.128).

In high-beta, inter-burst interval variability showed a trend (*p = *0.084; FDR *p = *0.338), with the PD-only adjusted comparison between PDFOG+ and PDFOG− also approaching significance before correction (β = 0.019, adjusted *p = *0.057; adjusted FDR *p = *0.227).

Topographic maps of secondary metrics (Fig. S5) revealed localized effects, particularly for theta amplitude and low-beta duration, but these did not show consistent corrected patterns across channels.

### Associations with FOG severity

**Fig. 2:**
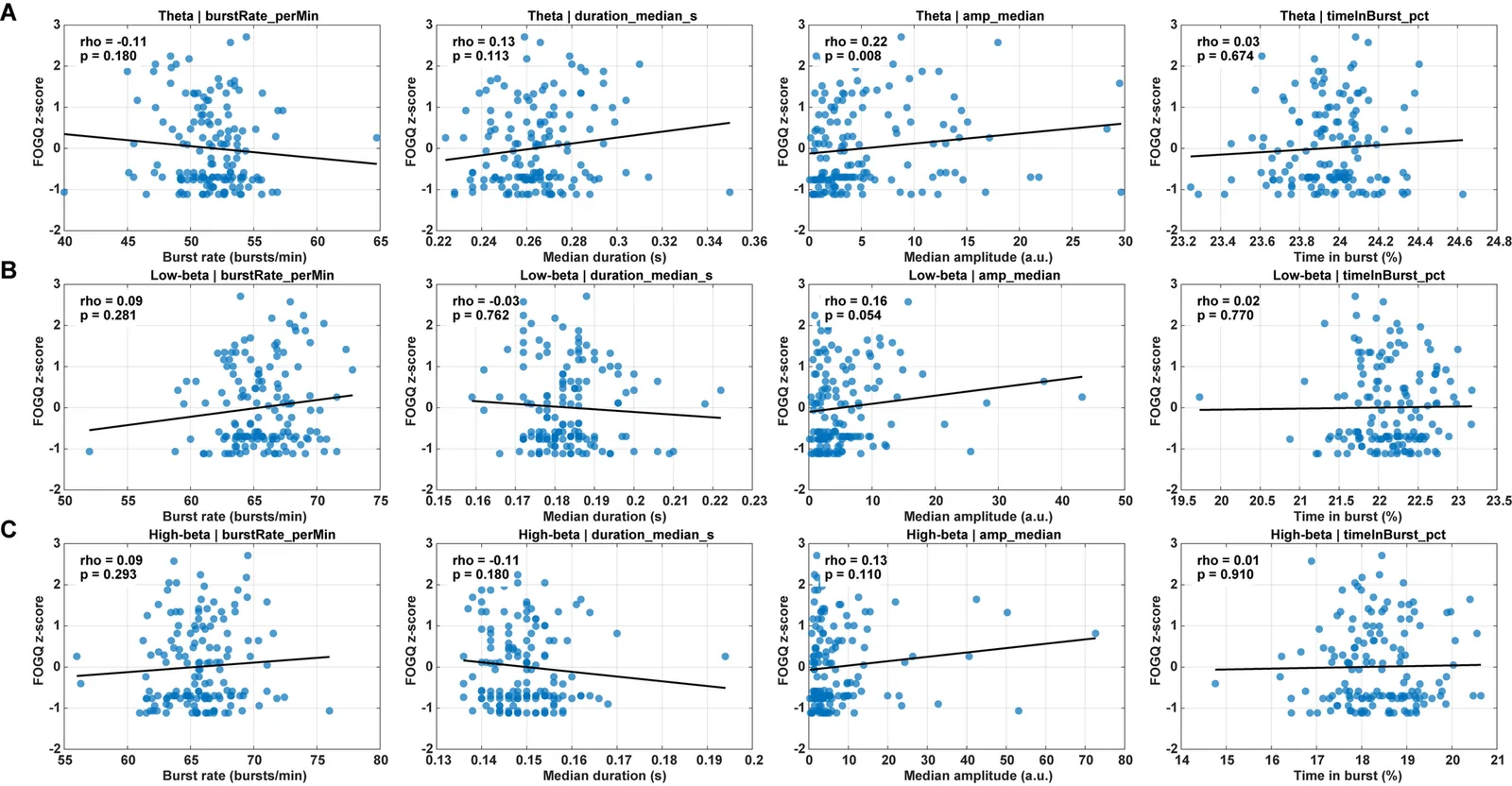
Associations between FOG severity and primary mid-frontal burst metrics. Scatter plots show correlations between center-normalized FOGQ z-scores and primary mid-frontal ‘Cz’ burst metrics in PD participants. Panels A, B and C show theta, low-beta, and high-beta bands, respectively. From left to right: each row shows burst rate, median burst duration, median burst amplitude, and time in burst. Theta median burst amplitude showed the strongest association with FOG severity among the primary metrics. Spearman’s rho and uncorrected p-values are displayed within each plot. FDR-corrected p-values are reported in Table S4

FOG severity correlations (Fig. 2; Table S4) showed that theta burst amplitude was significantly associated with freezing severity. Median theta amplitude at mid-frontal Cz was positively correlated with FOGQ z-score (rho = 0.22, *p = *0.008, FDR *p = *0.032). Similarly, theta 90th percentile amplitude was significantly associated with severity (rho = 0.22, *p = *0.007, FDR *p = *0.028). No beta-band metrics were significantly associated with FOG severity after correction. Low-beta median amplitude showed a trend (rho = 0.16, *p = *0.054, FDR *p = *0.216), along with several secondary metrics that did not survive correction. Overall, these findings indicate that, among PD participants assessed in the clinically defined ON-medication state, FOG severity was most consistently linked to theta burst amplitude, whereas low-beta burst abnormalities primarily distinguished PD participants from healthy controls rather than reflecting continuous FOG severity.

## Discussion

This study investigated resting-state EEG burst dynamics in a large multi-site cohort of healthy controls and individuals with PD, with all PD participants assessed in the clinically defined ON-medication state. Three main findings emerged from the analyses. First, participants with PD exhibited altered low-beta burst dynamics at the mid-frontal Cz electrode, characterized by a higher burst rate and shorter median burst duration compared with healthy controls, and these effects were supported by consistent topographic patterns. Second, although low-beta burst rate and duration differed between healthy control and PD groups, adjusted analyses that accounted for disease duration and motor severity did not reveal significant differences between PDFOG+ and PDFOG− subgroups, suggesting that these abnormalities are more strongly related to PD status than to FOG specifically. Third, FOG severity was positively associated with theta amplitude-related burst metrics, including both median and high-percentile burst amplitude, indicating a dissociation whereby beta-band abnormalities reflect general motor network dysfunction, while theta-band amplitude relates more specifically to FOG severity.

In this study, the focus on the mid-frontal Cz electrode was driven by both methodological and physiological considerations. The mid-frontal Cz electrode was consistently available across all sites, enabling harmonized cross-site analysis, and its location is also relevant to lower limb control and gait [7, 19, 35]. It is important to note that the low-beta effects were not confined to mid-frontal Cz location, but were observed across several central and posterior electrodes. This distributed pattern is consistent with evidence that beta bursts represent network-level events rather than isolated local oscillations. Simultaneous subthalamic nucleus (STN) local field potentials (LFPs) and cortical EEG recordings have demonstrated that beta bursts are accompanied by increased temporal overlap and phase synchronization across the basal ganglia–cortical motor circuit, with stronger interregional overlap during longer bursts [43]. Similarly, magnetoencephalography activity time-locked to STN beta bursts has been identified across motor, somatosensory, associative, limbic, and other cortical regions, indicating that subthalamic beta events participate in distributed cortical network dynamics [44]. Nevertheless, because this study used an 11-channel montage, the topographic findings should be interpreted as evidence of distributed sensor-level involvement rather than precise anatomical localization.

The most robust findings were observed in the low-beta band, where participants with PD, assessed in the clinically defined ON-medication state, demonstrated more frequent, but shorter bursts, without differences in burst amplitude or total time in burst. This pattern indicates that PD is associated with altered temporal structuring of beta activity rather than simply increases in oscillatory power. This interpretation is consistent with recent perspectives emphasizing that beta activity occurs in transient bursts rather than continuous oscillations, and that burst timing characteristics may provide more meaningful insight into neural function than traditional spectral measures [45].

Prolonged beta bursts have been associated with PD pathophysiology in invasive STN LFP recordings, particularly in the OFF-medication state [15, 46]. However, the present cortical EEG findings were obtained at rest during the ON-medication state and, therefore, may reflect a different pattern of beta-burst dynamics. Specifically, the increased burst rate with shorter duration may indicate altered temporal organization of cortical beta activity, including potential fragmentation or rapid termination of prolonged beta synchronization, potentially influenced by dopaminergic modulation. Thus, this finding should be distinguished from the previous findings that have shown association between prolonged STN beta bursts and greater motor impairment [15, 26]. In the absence of simultaneous cortical-subthalamic recordings and direct ON–OFF medication comparisons, it remains unclear whether the observed pattern is attributable to residual disease-related dysfunction, dopaminergic modulation of beta activity, compensatory cortical reorganization, or overlapping contributions from these mechanisms.

Although low-beta burst rate and duration differed across the three groups, adjusted comparisons did not reveal significant differences between participants in the PDFOG+ and PDFOG− subgroups. These results suggest that resting low-beta abnormalities are more closely related to PD status and overall motor-network dysfunction than to FOG, specifically. This interpretation is compatible with the established involvement of beta synchronization in bradykinesia, rigidity, and reduced motor flexibility [7–9]. In contrast, FOG is a complex and episodic phenomenon that involves not only motor impairment, but also cognitive, attentional, and locomotor network dysfunction [3, 4, 32]. As a result, resting-state beta burst measures may capture baseline motor system abnormalities, but may not fully reflect the dynamic and context-dependent mechanisms that give rise to freezing episodes during gait, turning, or dual-task conditions. The absence of subgroup effects after adjustment further indicates that some apparent FOG-related differences may be attributable to disease duration and overall motor severity.

In contrast, theta burst amplitude was positively associated with continuous FOG severity. Both median and 90th-percentile theta burst amplitude correlated with within-site standardized FOGQ scores and survived correction for multiple comparisons. This observation is consistent with previous evidence implicating theta-band activity in cognitive control and conflict monitoring processes, particularly in mid-frontal cortical regions [35, 39].

FOG frequently occurs in situations requiring increased executive control, such as navigating complex environments or performing dual tasks, and prior studies have reported abnormal theta activity during the evolution of FOG episodes, as well as altered coupling between cortical cognitive networks and subcortical motor circuits [4, 5, 20]. One interpretation of these findings is that increased theta burst amplitude reflects greater recruitment of cognitive control mechanisms in individuals with more severe FOG, potentially as a compensatory response to impaired automatic motor control. Alternatively, abnormal theta activity may reflect maladaptive network instability or excessive conflict monitoring that contributes to freezing. Because the data were collected at rest and are cross-sectional, these interpretations cannot be definitively distinguished. Notably, theta burst amplitude was associated with continuous FOG severity, but did not distinguish between PDFOG+ and PDFOG− groups in adjusted models, suggesting that continuous clinical measures may be more sensitive to underlying neural dysfunction than categorical classifications.

Finally, secondary burst metrics did not reveal additional group differences after correction. However, theta 90th percentile amplitude remained significantly associated with FOG severity, reinforcing the interpretation that theta amplitude-related burst features are relevant to FOG status. The exploratory topographic analyses revealed spatially heterogeneous patterns, particularly for low-beta duration and theta amplitude measures, but these findings were less consistent than the primary mid-frontal Cz results and should be considered hypothesis-generating.

This study has several strengths, including its large multi-site cohort, inclusion of participants with both PDFOG− and PDFOG+ status, and harmonized analysis across different EEG acquisition systems. The use of burst-based metrics also extends conventional spectral analyses by characterizing the temporal structure of oscillatory activity. However, several limitations should be acknowledged. First, harmonization to an 11-channel montage improved cross-site consistency, but limited spatial resolution and precluded source localization. Second, resting-state recordings may have reduced sensitivity to the dynamic neural processes that occur during gait, turning, and freezing episodes. Third, all PD participants were assessed in the clinically defined ON-medication state, and the timing of the last dopaminergic medication dose was not uniformly standardized or recorded across sites. Previous studies have shown that dopaminergic medication can modulate beta power and beta-burst patterns [15, 16, 24]; therefore, the present results cannot determine whether the observed low-beta burst timing abnormalities reflect PD-related cortical network dysfunction, medication-related modulation, compensatory changes, or a combination of these mechanisms. Disease-stage information, including Hoehn and Yahr stage, was also not consistently available across all sites, limiting our ability to examine whether beta-burst abnormalities vary by disease stage. Future studies should incorporate standardized medication timing, direct ON–OFF medication comparisons, harmonized disease-stage assessment, and simultaneous cortical–subthalamic recordings to clarify the mechanistic basis of these findings. Fourth, FOG severity was measured using different instruments across sites, requiring within-site normalization. Fifth, bursts were operationally defined using an amplitude threshold. Although the 75th percentile threshold approach is consistent with prior beta-burst studies, threshold crossings should not be assumed to represent discrete biological events in every instance. Finally, the cross-sectional design prevents causal inference.

## Conclusion

In this large multi-site cohort study, resting-state EEG burst analysis revealed frequency-specific alterations in PD and FOG during the ON-medication state. PD participants showed more frequent, but shorter low-beta bursts compared with healthy controls, suggesting altered temporal organization of cortical beta activity during the medicated state. These abnormalities were primarily related to PD status rather than adjusted FOG subgroup status. In contrast, theta burst amplitude was associated with FOG severity and may reflect cognitive-motor network dysfunction relevant to freezing. These findings support burst-based EEG metrics as complementary markers of PD motor-network dysfunction and FOG severity and motivate future studies incorporating medication manipulations, higher-density recordings, and freezing provoking tasks.

## Supplementary Information

Below is the link to the electronic supplementary material.

Supplementary file1 (DOCX 1500 kb)

## Data Availability

Deidentified data can be requested from the authors.
