# Supplementary material for: Frequency-Specific EEG burst dynamics in Parkinson’s Disease and freezing of gait
Source: J Neurol. 2026 Sep 23;273(10):616. doi: 10.1007/s00415-026-14149-6 (PMC13601153; doi:10.1007/s00415-026-14149-6)
Supplement: Supplementary file 1 [file 415_2026_14149_MOESM1_ESM.docx]

**Supporting Information**

**Frequency-Specific EEG Burst Dynamics in Parkinson’s Disease and Freezing of Gait**

**Table S1: Demographic and clinical characteristics by study site.**

**Site 1**

| **Measure** | **HC** | **PD** | **PDFOG**− | **PDFOG+** | **HC vs. PD** | **PDFOG**− **vs. PDFOG+** | **HC vs. PDFOG**− **vs. PDFOG+** |
| --- | --- | --- | --- | --- | --- | --- | --- |
| Gender (M/F) | 23/18 | 58/25 | 27/14 | 31/11 | - | - | - |
| Age (years) | 71.3 ± 1.2 | 68.6 ± 0.9 | 68.2 ± 1.2 | 69.0 ± 1.3 | p = 0.070 | p = 0.666 | p = 0.183 |
| DD (years) | - | 5.1 ± 0.4 | 4.2 ± 0.5 | 5.9 ± 0.7 | - | p = 0.052 | - |
| LEDD (mg) | - | 852.3 ± 50.9 | 722.3 ± 64.5 | 979.2 ± 74.0 | - | **p = 0.011*** | - |
| FOG-Q | - | 6.3 ± 0.6 | 1.6 ± 0.2 | 11.0 ± 0.6 | - | **p<0.001**** | - |
| MDS-UPDRS III | - | 13.4 ± 0.8 | 9.5 ± 0.9 | 17.2 ± 1.0 | - | **p < 0.001**** | - |
| MoCA | 26.6 ± 0.3 | 24.4 ± 0.4 | 25.3 ± 0.5 | 23.6 ± 0.6 | **p < 0.001**** | **p = 0.029*** | **p < 0.001**** |

**Site 2**

| Gender (M/F) | 9/18 | 22/10 | 10/7 | 12/3 | - |  | - |
| --- | --- | --- | --- | --- | --- | --- | --- |
| Age (years) | 65.3 ± 1.4 | 70.6 ± 1.5 | 68.9 ± 2.3 | 72.5 ± 1.9 | **p = 0.015*** | p = 0.241 | **p = 0.026*** |
| DD (years) | - | 5.7 ± 0.6 | 4.0 ± 0.7 | 7.6 ± 0.8 | - | **p = 0.003**** | - |
| LEDD (mg) | - | 554.8 ± 92.4 | 429.8 ± 129.9 | 706.5 ± 123.4 | - | p = 0.133 | - |
| FOG-Q | - | 6.4 ± 1.1 | 1.9 ± 0.7 | 11.6 ± 1.1 | - | **p<0.001**** | - |
| MDS-UPDRS III | - | 27.8 ± 3.3 | 21.2 ± 3.3 | 35.3 ± 5.3 | - | **p = 0.033*** | - |
| MoCA | 26.9 ± 0.5 | 24.7 ± 0.7 | 25.9 ± 0.8 | 23.3 ± 1.2 | **p = 0.011*** | p = 0.080 | **p = 0.005**** |

**Site 3**

| Gender (M/F) | 10/10 | 18/16 | 9/9 | 9/7 | - |  | - |
| --- | --- | --- | --- | --- | --- | --- | --- |
| Age (years) | 69.3 ± 1.8 | 70.0 ± 1.2 | 70.5 ± 1.0 | 69.5 ± 2.3 | p = 0.735 | p = 0.694 | p = 0.870 |
| DD (years) | - | 6.9 ± 0.8 | 5.4 ± 0.7 | 8.5 ± 1.5 | - | p = 0.083 | - |
| LEDD (mg) | - | 711.4 ± 67.2 | 545.4 ± 75.4 | 910.7 ± 95.9 | - | **p = 0.006**** | - |
| NFOG-Q | - | 6.6 ± 1.6 | 0.0 ± 0.0 | 13.9 ± 2.3 | - | - | - |
| MDS-UPDRS III | - | 32.1 ± 2.6 | 26.4 ± 3.0 | 38.4 ± 3.8 | - | **p = 0.019*** | - |
| MoCA | 27.4 ± 0.4 | 27.7 ± 0.4 | 27.6 ± 0.6 | 27.8 ± 0.5 | p = 0.580 | p = 0.793 | p = 0.838 |

Data are presented as mean ± SEM. Group differences between HC and participants with PD were evaluated using independent t-tests Group differences between PDFOG− and PDFOG+ were also evaluated using independent t-tests. Comparisons among HC, PDFOG**–**, and PDFOG+ groups were evaluated using one-way ANOVA.

Statistical significance for t-tests and ANOVA presented as p-value. *p<0.05; **p<0.01

Abbreviations: DD, Disease duration; LEDD, Levodopa equivalent daily dose;

FOG-Q, Freezing of Gait Questionnaire; NFOG-Q, New Freezing of Gait Questionnaire; MDS-UPDRS III, motor Unified Parkinson’s Disease Rating Scale; MoCA, Montreal Cognitive Assessment; HC, healthy controls; PDFOG−, PD without freezing of gait; PDFOG+, PD with freezing of gait

**Table S2. Secondary mid-frontal Cz burst metrics in healthy controls and people with PD.**

| **Metric** | **Low-beta HC**  **(n = 88)** | **Low-beta PD**  **(n = 149)** | **p** | **pFDR** | **High-beta HC**  **(n = 88)** | **High-beta PD**  **(n = 149)** | **p** | **pFDR** |
| --- | --- | --- | --- | --- | --- | --- | --- | --- |
| 90^th^ percentile burst duration(s) | 0.324 | 0.318 | **0.021*** | 0.084 | 0.254 | 0.253 | 0.665 | 0.832 |
| 90^th^ percentile burst amplitude | 19.365 | 15.893 | 0.350 | 0.467 | 17.974 | 18.69 | 0.264 | 0.528 |
| Inter-burst interval CV | 0.725 | 0.722 | 0.754 | 0.754 | 0.768 | 0.757 | 0.261 | 0.528 |
| Burstiness index | 0.921 | 0.919 | 0.103 | 0.206 | 0.925 | 0.925 | 0.832 | 0.832 |

Data are presented as mean ± SEM of participant-level burst metrics. Group differences between HC and participants with PD were evaluated using independent t-tests. Reported p-values are uncorrected. pFDR values represent Benjamini-Hochberg false discovery rate-adjusted p-values corrected within each frequency band across secondary burst metrics.

Statistical significance was defined as *pFDR < 0.05.

Abbreviations: HC, healthy control; PD, Parkinson’s disease; SEM, standard error of the mean; CV, coefficient of variation.

**Table S3. Secondary mid-frontal Cz burst metrics across healthy controls, PDFOG-, and PDFOG+ groups.**

| **Metric** | **HC**  **(n = 88)** | **PDFOG**−  **(n = 76)** | **PDFOG+**  **(n = 73)** | **p** | **pFDR** | **Adjusted β**  **(PDFOG**− **vs. PDFOG+)** | **p** | **Adjusted pFDR** |
| --- | --- | --- | --- | --- | --- | --- | --- | --- |
| **Theta** |  |  |  |  |  |  |  |  |
| 90th percentile burst duration(s) | 0.43 ± 0.003 | 0.44 ± 0.004 | 0.43 ± 0.003 | 0.507 | 0.507 | -0.008 | 0.190 | 0.439 |
| 90th percentile burst amplitude | 58.09 ± 38.21 | 13.80 ± 1.79 | 16.86 ± 2.79 | **0.021*** | 0.086 | -1.678 | 0.625 | 0.625 |
| Inter-burst interval CV | 0.65 ± 0.005 | 0.64 ± 0.005 | 0.649 ± 0.005 | 0.280 | 0.373 | 0.007 | 0.411 | 0.549 |
| Burstiness index | 0.89 ± 0.001 | 0.89 ± 0.002 | 0.89 ± 0.001 | 0.193 | 0.373 | -0.003 | 0.219 | 0.439 |
| **Low-beta** |  |  |  |  |  |  |  |  |
| 90th percentile burst duration(s) | 0.32 ± 0.002 | 0.32 ± 0.003 | 0.32 ± 0.002 | 0.032* | 0.128 | -0.006 | 0.177 | 0.639 |
| 90th percentile burst amplitude | 19.37 ± 4.05 | 14.69 ± 2.77 | 17.28 ± 2.28 | 0.398 | 0.531 | 1.202 | 0.756 | 0.756 |
| Inter-burst interval CV | 0.73 ± 0.005 | 0.72 ± 0.006 | 0.73 ± 0.005 | 0.928 | 0.928 | 0.003 | 0.751 | 0.756 |
| Burstiness index | 0.92 ± 0.001 | 0.92 ± 0.001 | 0.92 ± 0.002 | 0.188 | 0.376 | -0.002 | 0.320 | 0.639 |
| **High-beta** |  |  |  |  |  |  |  |  |
| 90th percentile burst duration(s) | 0.25 ± 0.002 | 0.25 ± 0.002 | 0.25 ± 0.002 | 0.781 | 0.781 | 0.001 | 0.841 | 0.841 |
| 90th percentile burst amplitude | 17.97 ± 3.97 | 17.20 ± 4.10 | 20.41 ± 4.29 | 0.335 | 0.670 | 1.844 | 0.774 | 0.841 |
| Inter-burst interval CV | 0.77 ± 0.005 | 0.75 ± 0.007 | 0.77 ± 0.006 | 0.084 | 0.338 | 0.019 | 0.057 | 0.227 |
| Burstiness index | 0.93 ± 0.001 | 0.92 ± 0.001 | 0.93 ± 0.001 | 0.755 | 0.781 | 0.001 | 0.595 | 0.841 |

Data are presented as mean ± SEM of participant-level burst metrics. Group differences were assessed using Kruskal-Wallis tests. Reported p-values are uncorrected. pFDR values represent Benjamini-Hochberg false discovery rate-adjusted p-values corrected within each frequency band across the four secondary burst metrics. β coefficients represent the estimated PDFOG+ versus PDFOG− effect derived from linear models restricted to participants with Parkinson disease and adjusted for disease duration and MDS-UPDRS III score. Adjusted pFDR values represent false discovery rate-adjusted p-values for the adjusted models.

Statistical significance was defined as *pFDR < 0.05.

Abbreviations: HC, healthy controls; PD, Parkinson’s disease PDFOG−, PD without freezing of gait; PDFOG+, PD with freezing of gait; SEM, standard error of the mean; CV, coefficient of variation; a.u., arbitrary units; MDS-UPDRS III, Movement Disorder Society Unified Parkinson's Disease Rating Scale Part III.

**Table S4: Correlations between FOGQ Z-scores and mid-frontal Cz burst metrics.**

| **Metric** | **Theta r** | **Theta p** | **Theta pFDR** | **Low-beta r** | **Low-beta p** | **Low-beta pFDR** | **High-beta r** | **High-beta p** | **High-beta pFDR** |
| --- | --- | --- | --- | --- | --- | --- | --- | --- | --- |
| Burst rate (bursts/min) | -0.111 | 0.180 | 0.240 | 0.089 | 0.281 | 0.562 | 0.087 | 0.293 | 0.390 |
| Median burst duration(s) | 0.131 | 0.113 | 0.225 | -0.025 | 0.762 | 0.770 | -0.111 | 0.180 | 0.360 |
| Median burst amplitude | 0.216 | **0.008*** | **0.033*** | 0.158 | 0.054 | 0.218 | 0.132 | 0.110 | 0.360 |
| Time in burst (%) | 0.035 | 0.674 | 0.674 | 0.024 | 0.770 | 0.770 | 0.009 | 0.910 | 0.910 |
| 90^th^ percentile burst duration(s) | -0.021 | 0.800 | 0.800 | -0.144 | 0.081 | 0.162 | 0.033 | 0.687 | 0.687 |
| 90^th^ percentile burst amplitude | 0.220 | **0.007*** | **0.029*** | 0.160 | 0.052 | 0.162 | 0.165 | 0.046 | 0.183 |
| Inter-burst interval CV | 0.081 | 0.328 | 0.438 | 0.097 | 0.242 | 0.323 | 0.120 | 0.146 | 0.293 |
| Burstiness index | -0.134 | 0.104 | 0.209 | -0.080 | 0.333 | 0.333 | 0.052 | 0.527 | 0.687 |

Spearman rank correlations were calculated between FOGQ Z-scores and participant-level Cz burst metrics. Reported p-values are uncorrected. pFDR values represent Benjamini-Hochberg false discovery rate-adjusted p-values corrected within each frequency band across the eight burst metrics.

Statistical significance was defined as *pFDR < 0.05.

Abbreviations: FOGQ, Freezing of Gait Questionnaire; CV, coefficient of variation.

**Figure S1. Topographic distribution of primary beta-band burst differences between healthy controls and participants with PD.** Topographic maps illustrating differences between healthy controls (HC) and participants with Parkinson disease (PD) across the 11 common EEG channels for the four primary burst metrics: burst rate, median burst duration, median burst amplitude, and time in burst. Difference maps are displayed as PD minus HC. Panel A presents low-beta results, and Panel B presents high-beta results. Low-beta maps demonstrated the most pronounced group differences, characterized by higher burst rates and shorter median burst durations in PD relative to HC. Asterisks denote channels with uncorrected p < 0.05, whereas circles indicate channels that remained significant after false discovery rate (FDR) correction across the 11 channels within each frequency band–metric combination.

**Figure S2. Topographic distribution of primary theta- and alpha-band burst differences between healthy controls and PD participants.** Topographic maps illustrating differences between healthy controls (HC) and participants with Parkinson disease (PD) across all 11 EEG channels for the four primary burst metrics: burst rate, median burst duration, median burst amplitude, and time in burst. Difference maps are displayed as PD − HC. Panel A presents theta-band results, and Panel B presents alpha-band results. These analyses were exploratory and were included to visualize frequency-specific spatial patterns outside the primary beta-band hypothesis. Asterisks denote channels with uncorrected p < 0.05, whereas circles indicate channels that remained significant after false discovery rate (FDR) correction across the 11 channels within each frequency band–metric combination.

**Figure S3. Topographic distribution of secondary burst differences between healthy controls and participants with PD across frequency bands.** Topographic maps illustrating differences between healthy controls (**HC**) and participants with Parkinson’s disease (PD) across all 11 EEG channels for the four secondary burst metrics: 90th percentile burst duration, 90th percentile burst amplitude, inter-burst interval coefficient of variation, and burstiness index. Difference maps are displayed as PD − **HC**. Panels A–D present theta-, alpha-, low-beta-, and high-beta-band results, respectively. These analyses were exploratory and were included to assess whether secondary burst characteristics exhibited frequency-specific spatial patterns of group differences beyond the primary burst metrics. Asterisks denote channels with uncorrected p < 0.05, whereas circles indicate channels that remained significant after false discovery rate (FDR) correction across the 11 channels within each frequency band–metric combination.

**Figure S4. Topographic distribution of primary burst differences across healthy control, PDFOG−, and PDFOG+ groups.** Topographic maps illustrating healthy control (HC), PDFOG+, and PDFOG**−** group contrasts across the 11 common EEG channels for the four primary burst metrics: burst rate, median burst duration, median burst amplitude, and time in burst. Panels A–C present theta-, low-beta-, and high-beta-band results, respectively. Within each panel, difference maps are displayed for PDFOG− − HC, PDFOG+ − HC, and PDFOG+ − PDFOG−. For the PDFOG+ − PDFOG− contrast, color scales represent the unadjusted mean group difference, whereas significance markers reflect the adjusted PD-only group comparison controlling for disease duration and MDS-UPDRS III scores. Asterisks denote channels with uncorrected p < 0.05, whereas circles indicate channels that remained significant after false discovery rate (FDR) correction across the 11 channels within each frequency band–metric–contrast combination.

**Figure S5. Topographic distribution of secondary burst differences across HC, PDFOG−, and PDFOG+ groups.** Topographic maps illustrating healthy control (HC), PDFOG+, and PDFOG**−** group contrasts across the 11 common EEG channels for the four secondary burst metrics: 90th percentile burst duration, 90th percentile burst amplitude, inter-burst interval coefficient of variation, and burstiness index. Panels A–C present theta-, low-beta-, and high-beta-band results, respectively. Within each panel, difference maps are displayed for PDFOG− − HC, PDFOG+ − HC, and PDFOG+ − PDFOG−. For the PDFOG+ − PDFOG− contrast, color scales represent the unadjusted mean group difference, whereas significance markers reflect the adjusted PD-only group comparison controlling for disease duration and MDS-UPDRS III scores. These analyses were exploratory and were included to assess whether secondary burst characteristics exhibited frequency-specific spatial patterns of group differences beyond the primary burst metrics. Asterisks denote channels with uncorrected p < 0.05, whereas circles indicate channels that remained significant after false discovery rate (FDR) correction across the 11 channels within each frequency band–metric–contrast combination.
